# Social Risk and Acute Health Care Utilization Among Insured Adults

**DOI:** 10.1001/jamanetworkopen.2025.4253

**Published:** 2025-04-01

**Authors:** Morgan Clennin, Mario Schootman, Emma L. Tucher, Liza M. Reifler, Suma Vupputuri, Meagan Brown, John Adams, Stacie L. Daugherty

**Affiliations:** 1 Institute for Health Research, Kaiser Permanente Colorado, Aurora; 2 Department of Health Systems Science, Kaiser Permanente Bernard J. Tyson School of Medicine, Pasadena, California; 3 Department of Medicine, College of Medicine, University of Arkansas for Medical Sciences, Little Rock; 4 Division of Research, Kaiser Permanente Northern California, Pasadena; 5 Mid-Atlantic Permanente Research Institute, Kaiser Permanente Mid-Atlantic States, Rockville, Maryland; 6 Kaiser Permanente Washington Health Research Institute, Seattle; 7 Department of Epidemiology, School of Public Health, University of Washington, Seattle; 8 Colorado Permanente Medical Group, Department of Cardiology, Denver; 9 Division of Cardiology, University of Colorado School of Medicine, Aurora

## Abstract

**Importance:**

Millions of Americans are impacted by adverse social risk factors such as financial strain, housing instability, and food insecurity. A better understanding of if and how these factors are associated with acute care utilization is needed.

**Objective:**

To examine the association between exposure to social risk factors and emergency department (ED) visits and hospitalizations among a sample of insured adults.

**Design, Setting, and Participants:**

This cohort study used US health data from a national initiative that employed a multistage, stratified sampling framework across 8 regional markets of an integrated health care delivery system. Eligible participants were insured adults who responded to health surveys. Population-based samples were taken proportionate to the sex and age distribution of each market.

**Exposures:**

Validated survey questions captured social risk exposure (positive screening for financial strain, housing instability, and/or food insecurity) during the past year; across 2 survey waves (2020, 2022). Self-reported social risk was categorized into mutually exclusive risk levels, social risk vs no social risk.

**Main Outcomes and Measures:**

Primary outcome was acute health care utilization defined as time to the first event (ED visit, hospitalization) observed following the first survey completion (January 2020 to July 2023). Weighted Cox proportional hazards regression examined the association between social risk and subsequent care utilization, adjusting for demographic and clinical covariates.

**Results:**

The analytic cohort included 9785 survey respondents. The weighted cohort data (mean age, 48.4 years [95% CI, 47.9-48.9 years]) included 54.1% female respondents (95% CI, 52.3%-55.9%); 14.6% of the sample were Asian (95% CI, 13.3%-16.0%), 8.1% Black (95% CI, 7.3%-9.1%), 27.1% Hispanic (95% CI, 25.5%-28.8%), and 43.6% non-Hispanic White (95% CI, 41.2%-44.7%); and 50.3% reported exposure to 1 or more social risk factor. During the follow-up period (median [IQR], 3.48 [3.01-3.50] years), 25.4% (95% CI, 22.9%-28.1%) and 10.3% (95% CI, 8.9%-11.9%) of the cohort experienced an ED visit and hospitalization, respectively. Utilization rates varied by level of social risk exposure. Respondents who reported any social risk had a 21% higher risk of an ED visit compared with those with no social risk exposure (adjusted hazard ratio [HR], 1.21 [95% CI, 1.03-1.41]). Social risk was not associated with hospitalizations (adjusted HR, 1.05 [95% CI, 0.84-1.32]).

**Conclusions and Relevance:**

In this cohort of 9785 adults, the significant association between social risk and time to first ED event warrants future study to determine if improved social risk are associated with lower ED utilization.

## Key Points

**Question:**

Is exposure to health-related social risk (financial strain, housing instability, food insecurity) associated with acute health care utilization (emergency department [ED] visits and hospitalizations) among a representative cohort of insured adults from an integrated health care delivery system?

**Findings:**

In this cohort study of US adults in a health care system, 25.4% and 10.3% of patients experienced an ED visit and hospitalization, respectively. Adults reporting social risk were significantly more likely to visit the ED (21%), but social risk was not associated with hospitalizations.

**Meaning:**

The association between elevated social risk and increased ED visits warrants future evaluation to determine if addressing these risks changes acute care utilization.

## Teaser

This cohort study of US adults in 8 regional health care markets examines the association of exposure to health-related social risk with emergency department visits and hospitalizations.

## Introduction

Social determinants of health (SDOH), the “conditions in the environments in which people are born, live, learn, work, play, worship, and age,”^1^ play a key role in health and disease.^2^ SDOH includes social risk factors, or conditions that may put an individual at higher risk for poor health. In the US, the most prevalent social risk factors are financial strain (eg, inability to pay for basic needs), housing instability (eg, inadequate housing and/or frequent moves), and food insecurity (eg, lack of access to sufficient and nutritious foods).^3,4,5,6,7,8,9^ These interlinked factors often generate a negative cycle wherein financial strain contributes to housing instability, perpetuating food insecurity and financial strain on households. Social risk exposure also increases the risk of adverse health outcomes and may be associated with increased health care utilization.^10,11,12,13,14,15,16,17^

Current research suggests an association between social risk exposure and utilization of acute health care services, including increased risk of emergency department (ED) visits and inpatient hospitalizations.^16,18,19^ Adults experiencing social risk may delay care for emerging health issues resulting in increased severity of health conditions and subsequently greater risk of ED utilization and hospitalization. Prior studies examining the association between social risk and acute care utilization have been limited by (1) a focus on a singular social risk factor, which limits ability to examine multiple interrelated factors simultaneously; (2) 1-time assessment of social risk exposure, which does not account for potential changes over time; and (3) lack of methodological rigor in the study design (eg, convenience sampling, cross-sectional, single site or region). To address these limitations, we examined the association between social risk factors (financial strain, housing instability, and food insecurity) and subsequent acute care utilization (ED visits and hospitalizations) in a large, geographically diverse, integrated health care delivery system.

## Methods

Leveraging data from a longitudinal quality improvement project, we conducted secondary analysis to examine the association of social risk factors with ED visits and hospitalizations among adults insured by Kaiser Permanente (KP)—an integrated health system located in 8 regional markets in the US (Colorado, Georgia, Hawaii, Mid-Atlantic states, Northern California, Northwest, Southern California, and Washington). The project was deemed to not be human participants research by the KP Interregional institutional review board and a waiver of consent was not deemed necessary. This project followed the Strengthening the Reporting of Observational Studies in Epidemiology (STROBE) reporting guidelines for observational cohort studies.

### Data Sources

The KP National Social Needs Survey (NSNS) measured health-related social risk factors among a representative sample of KP members across. The survey included 31 questions focused on 4 domains: social risk, social need, health status, and sociodemographic characteristics. The multimodal survey (web, phone, paper) was self-administered in English and Spanish at 2 time points: 1.0 survey wave (January to September 2020) and 2.0 survey wave (June 2022 to February 2023). The NSNS survey used a stratified sampling framework with a target sample size of 10 000 respondents. A total of 10 226 adults completed the 1.0 survey (response rate, 23.3%). Approximately 2 years later, each living adult that completed the 1.0 survey was contacted to complete a second survey (10 119 respondents; response rate, 62%). Logistic regression was used to construct nonresponse weights (truncated at the 95th percentile). These weights were combined with design weights for analysis, allowing for results to be generalizable to the KP membership sampling frame. Details regarding the survey sampling frame and methodology have been previously published.^20^

### Analytic Cohort

To construct the analytic cohort, NSNS respondents that completed the initial survey were eligible (10 226 respondents). NSNS data were merged with electronic health records (EHR) using a unique member ID (100% match). EHR data from January 2019 through July 2023 were extracted and included the following domains: acute care utilization, membership enrollment periods, health outcomes, and demographics. Exclusion criteria included: (1) those not enrolled in health plan at time of the first survey completion (allowing time for utilization to occur; 30-day lapse in coverage permitted) (338 respondents); and (2) those with incomplete data for social risk variables or covariates (151 respondents). Exclusions ranged from 3.1% to 6.2% of the regional samples. The final analytic cohort included 9785 adults with complete data (eFigure in Supplement 1).

### Measures

#### Utilization Outcomes

Guided by Andersen’s Behavioral Model for Health Services Use and prior research, we assessed the following outcomes, exposures, and covariates.^21,22,23,24,25,26^ The primary outcome was acute health care utilization defined as time to the first event observed following 1.0 survey completion through July 2023 (allowing for culmination of delays in care and/or chronic health management); with (1) emergency department (ED) visits and (2) inpatient hospitalizations examined separately. Each unique encounter was categorized by the highest level of care (eg, hospitalized patients admitted through the ED were counted as inpatient hospitalization only). Nonemergency and planned utilization visits, including newborn labor and delivery, were excluded from outcomes (339 respondents [2.6%]).

#### Social Risk Exposures

The primary exposure was a composite measure of social risk defined as self-reported experience with financial strain, housing instability, and/or food insecurity during the past 12 months using validated survey instruments.^27,28,29,30,31,32^ Financial strain was determined using 2 questions that asked about difficulty paying for basic needs (“How hard is it for you to pay for the very basics like food, housing, medical care, and heating?” Responses included very hard, hard, somewhat hard, not very hard, and not hard at all) and if there was money left at the end of each month to make ends meet (“Thinking about the past 12 months, would you say that at the end of each month you generally ended up with….” Responses included “more than enough to make ends meet,” “some left over to make ends meet,” “just enough to make ends meet,” “almost enough to make ends meet,” and “not enough to make ends meet”). Housing instability was determined via 4 questions that assessed ability to pay mortgage or rent on time (“In the past 12 months, was there a time when you were not able to pay the mortgage or rent on time?” yes or no), number of places lived (“In the past 12 months, how many places have you lived?” 1, 2, or 3 or more), unsteady place to sleep (“In the past 12 months, was there a time when you did not have a steady place to sleep or slept in a shelter?” yes or no), and living situation (“What is your living situation today?” Responses included “you have a steady place to live”; “you have a steady place to live but are worried about losing it in the future”; “you do not have a steady place to live”). Food insecurity was assessed via 3 questions: “Within the past 12 months, you worried whether your food would run out before you got money to buy more” (never true, sometimes true, often true); “Within the past 12 months, the food you bought just didn’t last and you didn’t have money to get more” (never true, sometimes true, often true); “Within the past 12 months, it was hard for you to get enough healthy food” (never true, sometimes true, often true). Social risk was assessed at 2 time points (1.0 and 2.0 survey waves). The level of social risk exposure at each time point was determined by categorizing a respondent’s survey responses into 1 of 2 mutually exclusive levels (our composite social risk exposure): (1) no social risk: no reported experience with financial strain, housing instability, or food insecurity; (2) social risk: reporting any experience with one or more of the observed social risk factors (eTable 1 in Supplement 1 describes criteria used to define social risk exposure). In a secondary analysis, we examined each of the social risk factors individually (ie, financial strain, housing instability, food insecurity) and the count of social risks reported.

#### Covariates

Sociodemographic characteristics from the survey included age group (18 to 34 years, 35 to 64 years, 65 years or older), sex (female, male), self-identified race and ethnicity (Hispanic, non-Hispanic African American or Black, non-Hispanic Asian, non-Hispanic White, and other or multiple races selected), education level (high school degree or less, some college, 4 or more years of college), marital status (previously married, never married, married), insurance type (commercial, Medicaid, Medicare, dual), income level (below $35 000, $35 000 to $75 000, $75 000 and above), and employment status (employed, unemployed, student, disability, retired). Race and ethnicity were included because previous studies have shown race is associated with acute care utilization outcomes. Health-related covariates included survey-reported physical and mental health status (fair or poor, good, very good or excellent) and EHR-derived number of comorbidities (0, 1 , 2 and higher; per Charlson comorbidity index).^33,34^

### Statistical Analysis

All analyses were performed using weights that accounted for the complex sampling design and response probabilities for the 1.0 survey wave. Descriptive statistics and bivariate associations between utilization outcomes, social risk factors, and covariates were examined (ie, Rao-Scott χ^2^). ED and hospitalization rates by social risk status were estimated as least square means through Poisson models using 250 generated bootstrap subsamples and replicate weights; sum of squared deviations from replicate estimates were used to estimate rate standard errors and associated confidence intervals.^35^ All prevalence rates accounted for the stratification variables (region, sex, age, potential vulnerability of social risk) and complex survey design weights.

Weighted Cox proportional hazards regression was used to analyze the association between social risk exposure and subsequent care utilization. Baseline was assessed at the time of the first social needs survey (1.0) and follow-up occurred from the baseline until the first occurrence of the outcome (acute health care utilization). Censoring occurred at the time of patients’ death, disenrollment from their health system, or at the end of the observation period. Each Cox regression model incorporated social risk exposure as a time-varying covariate.^36^ If an individual responded to the second survey while still at risk (ie, no utilization since first survey and not censored), their social risk(s) may have changed, and new value(s) were used in estimating associations between social risks and outcomes for days at risk from the second survey forward. Composite social risk, housing instability, and food insecurity were assessed at both survey time points (time-varying covariate); financial strain was assessed using only the first survey due to changes in questions from the 1.0 to 2.0 surveys (fixed covariate) (eTable 1 in Supplement 1). Additionally, models included a flag for the COVID-19 pandemic (March 15, 2020, to August 31, 2020) due to shifts in utilization during this time.

A series of models were developed. First, we examined the unadjusted hazard ratio (HR) between social risk exposure and acute care utilization outcomes. Next, we examined the adjusted HR by adding all covariates regardless of their statistical significance in the adjusted model.^37,38,39^ To assess covariate collinearity, conditional indices of 3 or higher were examined and covariates with large variance decomposition proportion values were reparameterized or removed (ie, insurance type, income level, employment status, mental health status). Final adjusted models were rerun after removing or reformatting variables based on collinearity tests. Secondary analysis examined each social risk factor individually (ie, financial strain, housing instability, food insecurity) and number of social risk factors reported. Significance level of 2-sided *P* < .05 was used for all analyses. Analyses were conducted in SAS version 9.4 (SAS Institute Inc).

## Results

The analytic cohort included 9785 survey respondents (Table 1). Weighted baseline sample characteristics (mean age, 48.4 years [95% CI, 47.9-48.9 years]) included 54.1% female respondents (95% CI, 52.3%-55.9%); 14.6% of the sample were Asian (95% CI, 13.3%-16.0%), 8.1% Black (95% CI, 7.3%-9.1%), 27.1% Hispanic (95% CI, 25.5%-28.8%), and 43.6% White (95% CI, 41.2%-44.7%). In all, 13.9% (95% CI, 12.7%-15.2%) self-rated their physical health as fair to poor. Half (50.3%) of the weighted cohort screened positive for at least 1 social risk factor on the 1.0 survey. Individuals reporting social risk were significantly younger (ages 18 to 34 years: 34.1% [95% CI, 31.7%-36.5%] vs 22.3% [95% CI, 20.1%-24.6%]), more likely to identify as Hispanic ethnicity (34.8% [95% CI, 32.4%-37.2%] vs 19.4% [95% CI, 17.3%-21.7%]), had lower education attainment (high school graduate or General Educational Development accreditation: 28.6% [95% CI, 26.5%-30.9%] vs 15.0% [95% CI, 13.2%-16.9%]), never married (25.3% [95% CI, 23.2%-27.6%] vs 15.1% [95% CI, 13.3%-17.2%]), and reported poorer self-rated health status (fair or poor: 19.3% [95% CI, 17.4%-21.3%] vs 8.5% [95% CI, 7.2%-10.1%]) compared with those who did not report any social risk. At the 1.0 survey, the proportion (SE) of respondents that screened positive for individual social risk factors ranged from 16.2% (0.65) (housing instability) to 30.0% (0.82) (food insecurity) to 43.2% (0.89) (financial strain) (Table 2). Compared with those that met inclusion criteria (9785 respondents), those excluded (489 respondents) were more likely to be younger, have lower educational attainment, more often commercially insured, and screened positive for social risk (eTable 2 in Supplement 1).

**Table 1.** Weighted Baseline Cohort Descriptives

Response options	Weighted analytic cohort, % (95% CI)^a^	Any social risk exposure^b^		
		No, % (95% CI) (49.7%)	Yes, % (95% CI) (50.3%)	*P* value
Sociodemographic characteristics				
Age group, y				
18-34	28.2 (26.6-29.9)	22.3 (20.1-24.6)	34.1 (31.7-36.5)	<.001
35-64	52.5 (50.7-54.2)	53.0 (50.5-55.6)	51.9 (49.3-54.4)	
≥65	19.3 (18.0-20.7)	24.7 (22.7-26.8)	14.0 (12.5-15.7)	
Sex				
Female	54.1 (52.3-55.9)	51.7 (49.0-54.3)	56.4 (53.9-59.0)	.009
Male	45.9 (44.1-47.7)	48.3 (45.8-50.9)	43.6 (41.1-46.1)	
Race and ethnicity^c^				
African American or Black (non-Hispanic)	8.1 (7.3-9.1)	6.6 (5.4-7.8)	9.7 (8.4-11.1)	<.001
Asian (non-Hispanic)	14.6 (13.3-16.0)	14.8 (12.8-16.9)	14.4 (12.6-16.4)	
Hispanic, Latino/a, or Spanish origin	27.1 (25.5-28.8)	19.4 (17.3-21.7)	34.8 (32.4-37.2)	
White (non-Hispanic)	42.9 (41.2-44.7)	53.8 (51.2-56.3)	32.3 (30.1-34.5)	
Other, multiple races, or unknown	7.2 (6.2-8.2)	5.5 (4.3-6.9)	8.9 (7.5-10.5)	
Education level				
High school graduate, GED, or less	21.8 (20.4-23.3)	15.0 (13.2-16.9)	28.6 (26.5-30.9)	<.001
Some college or 2-y degree	30.9 (29.2-32.5)	27.0 (24.9-29.4)	34.6 (32.3-37.1)	
4-y College graduate or more	46.8 (45.0-48.6)	57.7 (55.1-60.2)	36.0 (33.6-38.5)	
Marital status				
Never married	20.3 (18.9-21.8)	15.1 (13.3-17.2)	25.3 (23.2-27.6)	<.001
Married or living with partner	65.6 (63.9-67.3)	72.0 (69.7-74.3)	59.3 (56.8-61.7)	
Previously married	13.8 (12.6-15.0)	12.5 (10.9-14.2)	15.0 (13.4-16.8)	
Insurance type				
Commercial	69.1 (67.5-70.6)	68.2 (65.9-70.5)	69.9 (67.7-72.1)	<.001
Medicare	17.9 (16.7-19.2)	22.8 (20.8-24.8)	13.2 (11.6-14.8)	
Medicaid	4.7 (4.1-5.2)	1.5 (1.1-2.0)	7.8 (6.8-8.9)	
Individual or dual	8.3 (7.4-9.4)	7.5 (6.2-9.0)	9.2 (7.9-10.6)	
Income level, $				
<35 000	20.2 (18.7-21.6)	6.73 (5.5-8.1)	27.8 (25.7-29.9)	<.001
35 000-75 000	31.6 (29.8-33.4)	21.5 (19.5-23.5)	32.7 (30.3-35.1)	
≥75 000	48.3 (46.4-50.2)	57.2 (54.7-59.7)	25.8 (23.6-28.2)	
Employment status				
Employed	66.5 (64.9-68.2)	63.6 (61.1-66.0)	69.5 (67.2-71.7)	<.001
Student (not employed)	2.7 (2.1-3.4)	2.3 (1.5-3.3)	3.2 (2.4-4.2)	
Unemployed, caregiver (not a student)	9.0 (8.0-10.0)	6.5 (5.3-7.9)	11.5 (10.0-13.1)	
Disability	2.6 (2.1-3.2)	1.0 (0.5-1.7)	4.2 (3.3-5.2)	
Retired	18.8 (17.5-20.1)	26.4 (24.3-28.6)	11.3 (9.9-12.8)	
Health status				
Self-rated physical health				
Excellent or very good	48.3 (46.5-50.1)	57.1 (54.6-59.7)	39.6 (37.1-42.1)	<.001
Good	37.7 (36.0-39.5)	34.3 (31.9-36.8)	41.1 (38.7-43.6)	
Fair or poor	13.9 (12.7-15.2)	8.5 (7.2-10.1)	19.3 (17.4-21.3)	
Self-rated mental health				
Excellent or very good	55.9 (54.1-57.7)	66.3 (63.9-68.7)	45.5 (43.0-48.0)	<.001
Good	29.6 (28.0-31.3)	25.3 (23.1-27.6)	33.9 (31.5-36.3)	
Fair or poor	14.5 (13.3-15.8)	8.3 (7.0-9.7)	20.6 (18.7-22.7)	
Charlson Comorbidity Index				
None	60.8 (59.0-62.5)	59.6 (57.1-62.1)	61.9 (59.4-64.6)	.26
1	21.1 (19.7-22.6)	22.3 (20.2-24.4)	20.0 (18.0-22.0)	
≥2	18.1 (16.9-19.5)	18.1 (16.3-20.0)	18.2 (16.4-20.0)	

Abbreviation: GED, General Educational Development.

^a^ Weighted sample, 7 665 119 respondents; unweighted sample, 9785 respondents.

^b^ Any social risk defined as screening positive for 1 or more social risk factors including housing instability, food insecurity, and/or financial strain (eTable 1 in Supplement 1).

^c^ Self-identified race and ethnicity categories were collapsed for analyses due to small sample size and power. Hispanic category was defined as individuals identifying as Hispanic, Latino/a or Spanish origin, or independently of the race group(s) selected. Other race category includes Pacific Islander (1.8%); American Indian or Alaska Native (0.4%); multiracial, with 2 or more groups selected (2.9%); and other race or unknown (0.2%).

**Table 2.** Patients Reporting Social Risk Exposure by NSNS Survey Wave

Social risk factors	Patients, weighted % (SE)	
	NSNS wave 1.0^a^	NSNS wave 2.0^b^
Any financial strain, housing instability, food insecurity^c^		
Social risk	50.3 (0.89)	48.8 (1.09)
No social risk	49.7 (0.89)	51.2 (1.09)
Financial strain^d^^,^^e^		
Social risk	43.2 (0.89)	57.0 (1.09)
No social risk	56.8 (0.89)	43.0 (1.09)
Housing instability^f^		
Social risk	16.2 (0.65)	13.8 (0.75)
No social risk	83.8 (0.65)	86.2 (0.75)
Food insecurity^g^		
Social risk	30.0 (0.82)	24.0 (0.94)
No social risk	70.0 (0.82)	76.0 (0.94)
Total social risk factors, No.		
3	10.9 (0.55)	8.8 (0.64)
2	17.3 (0.68)	13.8 (0.75)
1	22.1 (0.75)	26.2 (0.98)
None	49.7 (0.89)	51.2 (1.09)

Abbreviation: NSNS, National Social Needs Survey.

^a^ The 1.0 survey wave administered between January to September 2020, 9785 respondents met inclusion criteria and were included in the analytic study cohort (unweighted sample).

^b^ The 2.0 survey wave was administered between June 2022 to February 2023; 6003 of 9785 1.0 respondents completed the 2.0 survey (unweighted sample); weights accounted for study design and nonresponse probability.

^c^ Any social risk defined as screening positive for 1 or more social risk factors including housing instability, food insecurity, and/or financial strain (eTable 1 in Supplement 1).

^d^ Financial strain questions and response options in Methods section.

^e^ Financial strain was assessed using only the 1.0 survey due to changes in questions from the 1.0 to 2.0 surveys (fixed covariate).

^f^ Housing instability questions and response options in Methods section.

^g^ Food insecurity questions and response options in Methods section.

### Acute Care Utilization

The median (IQR) follow-up time from first survey completion to the first outcome event or censorship was 3.48 (3.01-3.50) years; with 2270 respondents (23.2%) in the analytic cohort disenrolling from their KP insurance plan before July 31, 2023 (end of observation period, averaging 6.7% disenrollment annually). During this timeframe, 25.4% and 10.3% of the weighted cohort experienced an ED visit and hospitalization, respectively. Rates of ED visits and hospitalizations varied by level of social risk exposure (Figure) (eTable 3 in Supplement 1). Among those reporting social risk exposure, rates of ED visits were 115.0 (95% CI, 104.3-126.8) visits per 1000 person-years compared with 86.9 (95% CI, 78.6-96.1) visits per 1000 person-years among those with no exposure. Similarly, those reporting social risk exposure also experienced higher observed rates of hospitalizations at 40.6 (95% CI, 35.2-46.9) hospitalizations per 1000 person-years compared with 33.8 (95% CI, 29.2-39.1) hospitalizations per 1000 person-years among those without social risk. Examining individual social risk factors, similar patterns in rates of ED visits and hospitalizations were observed.

**Figure.**
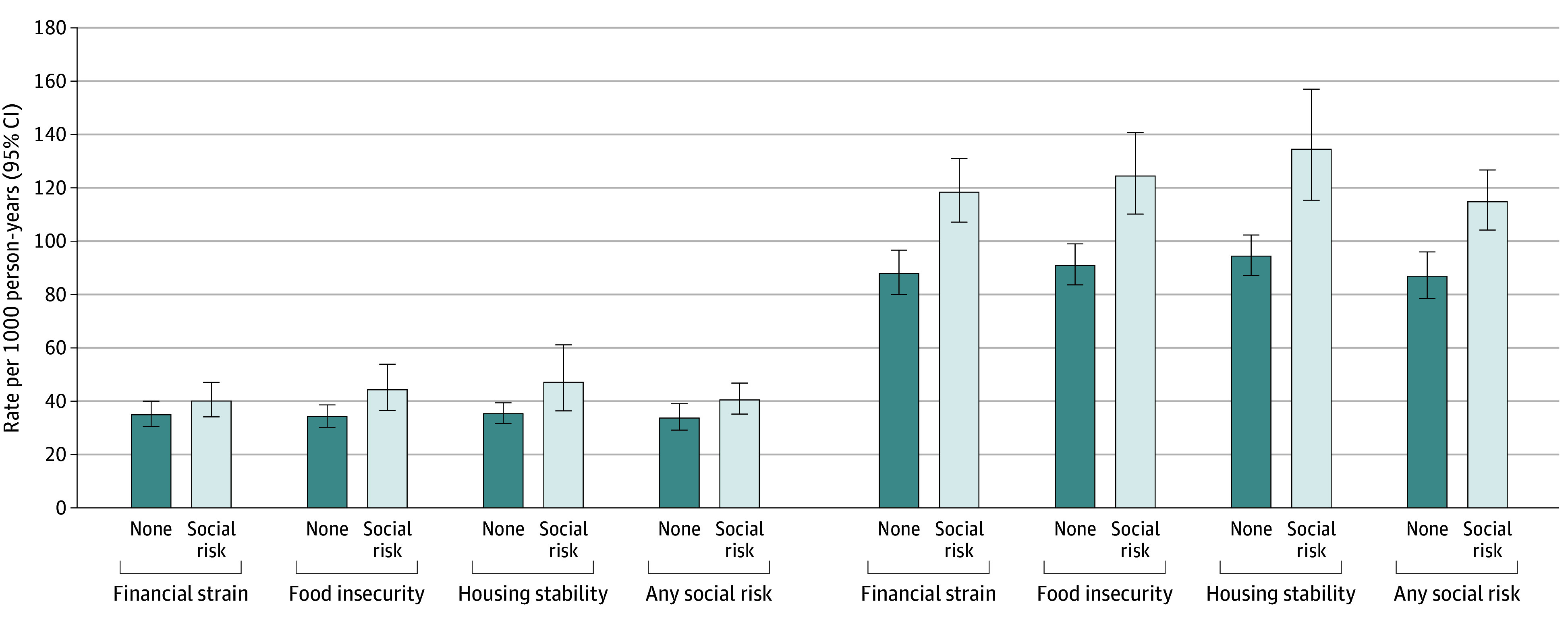
Rate of Acute Care Utilization (Emergency Department Visits and Hospitalizations) by Level of Social Risk Exposure

### Social Risk and ED Visits

In unadjusted models for the composite social risk exposure, social risk was associated with increased risk of ED visits (HR, 1.33 [95% CI, 1.16-1.53]) (Table 3). The association remained significant after adjustment (adjusted HR, 1.21 [95% CI, 1.03-1.41]). In secondary analysis examining individual social risk factors, adults that experienced housing instability (adjusted HR, 1.31 [95% CI, 1.09-1.57]), food insecurity (adjusted HR, 1.20 [95% CI, 1.02-1.40]), or financial strain (adjusted HR, 1.20 [95% CI, 1.03-1.39]) were significantly more likely to experience an ED visit than those without social risk. Additionally, the risk of experiencing an ED visit increased with the number of social risks reported (2 areas of risk: adjusted HR, 1.23 [95% CI, 1.001-1.51]; 3 areas of risk: adjusted HR, 1.45 [95% CI, 1.15-1.83]).

**Table 3.** Weighted Cox Regression: ED Visits by Social Risk Factors

Social risk factors	ED visits, HR (95% CI)	
	Unadjusted	Adjusted^a^^,^^b^
Any financial strain, housing instability, food insecurity		
No social risk	1 [Reference]	1 [Reference]
Social risk	1.33 (1.16-1.53)	1.21 (1.03-1.41)
Financial strain		
No social risk	1 [Reference]	1 [Reference]
Social risk	1.34 (1.17-1.54)	1.20 (1.03-1.39)
Housing instability		
No social risk	1 [Reference]	1 [Reference]
Social risk	1.36 (1.14-1.62)	1.31 (1.09-1.57)
Food insecurity		
No social risk	1 [Reference]	1 [Reference]
Social risk	1.37 (1.18-1.58)	1.20 (1.02-1.40)
Total social risk factors, No.		
None	1 [Reference]	1 [Reference]
1	1.17 (0.98-1.39)	1.11 (0.93-1.34)
2	1.40 (1.16-1.70)	1.23 (1.001-1.51)
3	1.63 (1.32-2.01)	1.45 (1.15-1.83)

Abbreviations: ED emergency department; HR, hazard ratios.

^a^ Model adjusted for individual-level sociodemographic factors (age group, sex, race and ethnicity, education, marital status) and health status factors (self-rated physical health, comorbidities). Covariates excluded from final analytic model after collinearity testing include insurance type, income level, employment status, self-reported mental health status.

^b^ Sensitivity analysis removing race and ethnicity from the final adjusted models were performed, findings remained consistent with no significant change in estimates (eTable 4 in Supplement 1).

### Social Risk and Inpatient Hospitalizations

In analysis of the association between the composite social risk exposure and hospitalization, the risk of experiencing a hospitalization was not significantly different among adults that reported social risk exposure compared with those reporting no social risk exposure (HR, 1.05 [95% CI, 0.84-1.32]) (Table 4). Similar nonsignificant findings were observed in secondary analysis of the individual social risk factor exposures and the total number of social risk factors reported.

**Table 4.** Weighted Cox Regression: Hospitalizations by Social Risk Factors

Social risk factors	Hospitalizations, HR (95% CI)	
	Unadjusted model	Adjusted model^a^^,^^b^
Any financial strain, housing instability, food insecurity		
No social risk	1 [Reference]	1 [Reference]
Social risk	1.18 (0.96-1.45)	1.05 (0.84-1.32)
Financial strain		
No social risk	1 [Reference]	1 [Reference]
Social risk	1.14 (0.93-1.41)	1.01 (0.81-1.26)
Housing instability		
No social risk	1 [Reference]	1 [Reference]
Social risk	1.30 (0.98-1.74)	1.28 (0.94-1.74)
Food insecurity		
No social risk	1 [Reference]	1 [Reference]
Social risk	1.22 (0.97-1.54)	1.05 (0.81-1.35)
No. of social risk areas (including financial strain, housing instability, and food insecurity)		
None	1 [Reference]	1 [Reference]
1	1.07 (0.83-1.39)	1.02 (0.79-1.32)
2	1.22 (0.92-1.62)	1.03 (0.76-1.41)
3	1.37 (0.95-1.99)	1.21 (0.80-1.83)

Abbreviation: HR, hazard ratio.

^a^ Model adjusted for individual-level sociodemographic factors (age group, sex, race and ethnicity, education, marital status) and health status factors (self-rated physical health, comorbidities). Covariates excluded from final analytic model after collinearity testing include insurance type, income level, employment status, self-reported mental health status.

^b^ Sensitivity analysis removing race and ethnicity from the final adjusted models were performed, findings remained consistent with no significant change in estimates (eTable 5 in Supplement 1).

## Discussion

Among a representative sample of nearly 10 000 insured adults from 8 regional markets of a US integrated health care delivery system, we found half of adults reported exposure to at least 1 social risk factor. Financial strain was the most reported social risk (43%), followed by food insecurity (30%) and housing instability (16%). Adults reporting social risk exposure were 21% more likely to experience an ED visit compared with those reporting no social risk exposure over a median follow-up of 3.5 years. When examining individual social risk factors, exposure to financial strain, housing instability, and food insecurity were independently associated with increased risks of ED visits. Exposure to multiple social risks concurrently was also associated with increased risk of ED visits. Despite similar observed patterns, social risk was not significantly associated with hospitalizations.

We observed a high prevalence of social risk exposure among a population of insured adults across the US. Our findings were roughly similar to other studies, although the prevalence estimates range considerably in prior research (37% to 64%).^40,41,42,43^ While varied, our findings add to the evidence suggesting that social risk is common, even among an insured population cared for in an integrated health care delivery system.^43^ Our findings also add to the developing understanding of the persistence of social risk exposure across time (eg, acute vs chronic exposure).^44,45^ Of those who responded to both surveys (approximately 1.5 to 2 years apart), we found 15.6% had a change in housing stability status and 20.8% had a change in food security status suggesting that social risk is not fixed and may evolve with some individuals moving in and out of risk (results not shown). Given the variability in social risk exposure over time, future studies should extend this work and capture the compounding and dynamic nature of social risk exposure to better understand the relationship between social risk exposure and acute care utilization, inform appropriate screening intervals, and test the appropriate window for social risk interventions.^44^

Similar to prior studies, we found an association between social risk and time to first ED utilization, including increased risk among those experiencing housing instability and food insecurity.^10,11,14,15,16,46,47,48,49,50,51^ Within the US health care system, the ED represents an essential source of care for acute, chronic, and emergent health issues. Although the prevalence of ED visits observed among our cohort was lower than others have reported (12.8% vs 22%), exposure to social risk factors remained associated with increased ED utilization.^52^ Inconsistent with prior research, our findings suggest that adults reporting social risk exposure were not more likely to be hospitalized.^19,53,54^ There are 2 potential factors that may explain this observed difference. First, the duration of the follow-up time may have been inadequate to capture hospitalizations, which are less prevalent than ED visits. Second, the prevalence of hospitalizations among our cohort was lower compared with national estimates among insured populations (4.3% vs 6.1%, respectively).^55^ It is possible our observed hospitalizations were underestimated due to failure to capture all admissions, particularly in regional markets that do not own their hospitals. However, examination of region-specific models in our prior work yielded similar nonsignificant results suggesting missing data are not a substantial contributor to these findings.^56^ Another plausible factor contributing to lower prevalence of hospitalizations is that patients with higher social risk may be more likely to seek ED care for nonacute conditions that do not require hospitalization. KP offers many options to receive timely follow-up after an ED visit. Hence, outpatient follow up care may be more accessible among patients that seek emergent care; thus, reducing the need for hospitalizations.^57,58^ Taken together, our findings and other studies suggest that social risk exposure is associated with higher ED utilization with mixed results on hospitalizations.

### Strengths and Limitations

Our findings expand the current research on social risk and acute care utilization in several ways. Specifically, this is one of the largest studies (8 regional markets of nearly 10 000 participants) with extended longitudinal follow-up (average of 3.5 years) to examine the association between social risks and subsequent acute care utilization—allowing for social risk to evolve over time.

However, some limitations should be noted. First, our representative sample of insured adults within an integrated health system may limit generalizability of these findings to broader populations (eg, uninsured or insured by different health plans). However, prior research has demonstrated that the demographic characteristics of KP’s population resemble the characteristics of the communities we serve.^59^ Second, it is possible that other unmeasured social risk factors and covariates may influence the observed findings; including insurance coverage type. We used validated survey instruments to assess 3 prevalent social risk exposures, extending prior research that has been predominately limited to single social risk exposures or inferring individual social risks based on area-level data (eg, deprivation indices).^60^ Additionally, our analytic approach allowed for updated social risk exposures data to be incorporated into model estimates as new data was available from the second survey. Other studies have typically been limited by examining social risk at a single point in time. Lastly, we cannot overlook the potential influence of the COVID-19 pandemic on utilization behavior. It is plausible that social risk exposure and utilization behavior were different during the initial months of the pandemic (which overlapped with the administration of the first survey wave). To help mitigate this issue, we adjusted for this expected difference in outcome as a precision variable in the model. Future studies are needed to extend these findings and better understand the evolution of social risk exposure over time including the frequency of such shifts and how combinations of social risk exposures influence acute care utilization (eg, lag time).

## Conclusions

Our results demonstrate an association between exposure to social risk factors and ED visits, showing how time-varying exposures to social risk may affect time to subsequent acute care utilization over a 3.5-year follow up period. As more health systems implement standardized social risk screening as part of their routine care, the availability and accessibility of patient-reported social risk exposure data will increase rapidly and provide a unique opportunity to expand upon these findings.^14,42,61,62,63,64,65,66,67,68^ If confirmed, our findings could be used to help prioritize interventions to address social risk—specifically those related to financial strain, housing instability, and food insecurity in the ED setting. Future studies will be required to assess the implementation of such health care–based interventions as well as their effectiveness in improving social risk exposure and acute care utilization outcomes.

## Notes

Supplement 1. **eTable 1.** Kaiser Permanente National Social Needs Survey: Social Risk Factors, Survey Questions, and Level of Social Risk Exposure Determination **eTable 2.** Weighted Baseline Cohort Descriptives Among the Analytic Study Cohort and Those Excluded **eTable 3.** Rate of Acute Care Utilization (ED Visits and Hospitalizations) by Level of Social Risk Exposure Among Weighted Analytic Cohort **eTable 4.** Sensitivity Analysis: Adjusted Hazard Ratios (HR) of Emergency Department (ED) Visits by Social Risk Factors; With and Without Race and Ethnicity Included in Final Adjusted Model **eTable 5.** Sensitivity Analysis: Adjusted Hazard Ratios (HR) of Hospitalizations by Social Risk Factors; With and Without Race and Ethnicity Included in Final Adjusted Model **eFigure.** CONSORT Diagram, Unweighted Cohort

Supplement 2.
Data Sharing Statement
