# Supplementary material for: Social Risk and Acute Health Care Utilization Among Insured Adults
Source: JAMA Netw Open. 2025 Apr 1;8(4):e254253. doi: 10.1001/jamanetworkopen.2025.4253 (PMC11962667; doi:10.1001/jamanetworkopen.2025.4253)
Supplement: Supplement 1. — eTable 1. Kaiser Permanente National Social Needs Survey: Social Risk Factors, Survey Questions, and Level of Social Risk Exposure Determination eTable 2. Weighted Baseline Cohort Descriptives Among the Analytic Study Cohort and Those Excluded eTable 3. Rate of Acute Care Utilization (ED Visits and Hospitalizations) by Level of Social Risk Exposure Among Weighted Analytic Cohort eTable 4. Sensitivity Analysis: Adjusted Hazard Ratios (HR) of Emergency Department (ED) Visits by Social Risk Factors; With and Without Race and Ethnicity Included in Final Adjusted Model eTable 5. Sensitivity Analysis: Adjusted Hazard Ratios (HR) of Hospitalizations by Social Risk Factors; With and Without Race and Ethnicity Included in Final Adjusted Model eFigure. CONSORT Diagram, Unweighted Cohort [file jamanetwopen-e254253-s001.pdf]

# Supplemental Online Content

Clennin M, Schootman M, Tucher EL, et al. Social risk and acute health care utilization among insured adults. *JAMA Netw Open.* 2025;8(4):e254253. doi:10.1001/jamanetworkopen.2025.4253

**eTable 1.** Kaiser Permanente National Social Needs Survey: Social Risk Factors, Survey Questions, and Level of Social Risk Exposure Determination

**eTable 2.** Weighted Baseline Cohort Descriptives Among the Analytic Study Cohort and Those Excluded

**eTable 3.** Rate of Acute Care Utilization (ED Visits and Hospitalizations) by Level of Social Risk Exposure Among Weighted Analytic Cohort

**eTable 4.** Sensitivity Analysis: Adjusted Hazard Ratios (HR) of Emergency Department (ED) Visits by Social Risk Factors; With and Without Race and Ethnicity Included in Final Adjusted Model

**eTable 5.** Sensitivity Analysis: Adjusted Hazard Ratios (HR) of Hospitalizations by Social Risk Factors; With and Without Race and Ethnicity Included in Final Adjusted Model

**eFigure.** CONSRT Diagram, Unweighted Cohort

This supplemental material has been provided by the authors to give readers additional information about their work.

© 2025 Clennin M et al. *JAMA Network Open*.

**eTable 1**. Kaiser Permanente National Social Needs Survey: Social risk factors, survey questions, and level of social risk exposure determination.

| Social Risk Factor Indicators | Social Risk Exposure Categories (Levels of Severity) | |
|---|---|---|
| | **No Risk** | **Social Risk** |
| **HOUSING INSTABILITY [1]** | | |
| Unable to pay the mortgage or rent on time<br>Yes<br>No | 'No' | 'Yes' |
| | ***AND*** | ***AND/OR*** |
| Number of places lived<br>One<br>Two<br>Three or more | 'one' ***OR*** 'two' | 'Three or more' |
| | ***AND*** | ***AND/OR*** |
| Unsteady Place to Sleep<br>Yes<br>No | 'No' | 'Yes |
| | ***AND*** | ***AND/OR*** |
| Living Situation<br>Have a steady place to live<br>Have a steady place to live but are worried about losing it in the future<br>Do not have a steady place to live | 'Have a steady place to live' | 'Have a steady place to live but are worried about losing it in the future'<br>***OR***<br>'Do not have a steady place to live' |

© 2025 Clennin M et al. *JAMA Network Open*.

| | | |
|---|---|---|
| **FOOD INSECURITY [2]** | | |
| Worried food would run out<br>Never true<br>Sometime true<br>Often true | 'Never true' | 'Somewhat true'<br>OR<br>'Often true' |
| | ***AND*** | ***AND/OR*** |
| Food did not last<br>Never true<br>Sometime true<br>Often true | 'Never true' | 'Somewhat true'<br>OR<br>'Often true' |
| | ***AND*** | ***AND/OR*** |
| Hard to get enough healthy food<br>Never true<br>Sometime true<br>Often true | 'Never true' | 'Somewhat true'<br>OR<br>'Often true' |
| **FINANCIAL STRAIN [3]** | | |
| Difficulty paying for basic needs<br>Very hard<br>Hard<br>Somewhat hard<br>Not very hard<br>Not hard at all | 'Not very hard'<br>OR<br>'Not hard at all' | 'Somewhat hard'<br>OR<br>'Hard'<br>OR<br>'Very hard' |
| | ***AND*** | ***AND/OR*** |
| Money left at end of each month to make ends meet | 'More than enough' | 'Just enough' |

© 2025 Clennin M et al. *JAMA Network Open*.

| | | |
|---|---|---|
| More than enough | OR<br><br>'Some left over' | OR<br><br>'Almost enough'<br><br>OR<br><br>'Not enough' |
| Some left over | | |
| Just enough | | |
| Almost enough | | |
| Not enough | | |
| **<u>SOCIAL RISK EXPOSURE (Composite Measure)</u>** | | |
| Housing Instability | 'No Risk' for all social risk factors | 'Social Risk'<br>for at least one social risk factor |
| Food Insecurity | | |
| Financial Strain | | |

[1] Housing Instability Questions (Response Options): #1: In the past 12 months, was there a time when you were not able to pay the mortgage or rent on time? (Yes, No): #2: In the past 12 months, how many places have you lived? (One, Two, Three or more); #3: In the past 12 months, was there a time when you did not have a steady place to sleep or slept in a shelter? (Yes, No); #4: What is your living situation today? (You have a steady place to live; you have a steady place to live but are worried about losing it in the future; you do not have a steady place to live)

[2] Food Insecurity Questions (Response Options): #1: Within the past 12 months, you worried whether your food would run out before you got money to buy more. (Never true; Sometimes true; Often true); #2: Within the past 12 months, the food you bought just didn't last and you didn't have money to get more. (Never true; Sometimes true; Often true); #3: Within the past 12 months, it was hard for you to get enough healthy food. (Never true; Sometimes true; Often true)

[3] Financial Strain Questions (Response Options): #1: How hard is it for you to pay for the very basics like food, housing, medical care, and heating? (Very hard, hard, somewhat hard, not very hard, not hard at all); #2: Thinking about the past 12 months, would you say that at the end of each month you generally ended up with…(More than enough to make ends meet, Some left over to make ends meet, Just enough to make ends meet, Almost enough to make ends meet, Not enough to make ends meet)

© 2025 Clennin M et al. *JAMA Network Open*.

**eTable 2**. Weighted baseline cohort descriptives among the analytic study cohort and those excluded.

| | Response Options | Analytic Cohort [1] | Excluded [2] | Significance |
|---|---|---|---|---|
| **Sociodemographic Characteristics** | | **% (95% CI) or Mean (SD)** | **% (95% CI) or Mean (SD)** | **Rao-Scott Chi-Square P-value** |
| Age Group | 18-34 years | 26.6% (25.0-28.2) | 44.3% (36.5-52.2) | <.0001 |
| | 35-49 years | 26.7% (25.0-28.4) | 15.3% (10.2-21.7) | |
| | 50-64 years | 26.3% (24.8-27.9) | 23.4% (17.6-30.2) | |
| | 65 + years | 20.4% (19.1-21.8) | 17.0% (12.0-23.0) | |
| Sex | Female | 54.1% (52.3-55.9) | 52.1% (44.3-59.9) | 0.6211 |
| | Male | 45.9% (44.1-47.7) | 47.9% (40.1-55.7) | |
| Race and Ethnicity | Hispanic, Latino/a or Spanish origin | 27.1% (25.5-28.8) | 27.2% (20.5-34.8) | 0.2510 |
| | White/Caucasian (non-Hispanic) | 43.6% (41.9-45.3) | 38.4% (31.1-46.1) | |
| | African American or Black (non-Hispanic) | 8.2% (7.3-9.1) | 11.7% (7.5-17.1) | |
| | Asian (non-Hispanic) | 15.9% (14.5-17.3) | 18.2% (12.5-25.2) | |
| | Pacific Islander (non-Hispanic) | 1.8% (1.3-2.4) | 1.6% (0.5-3.6) | |
| | American Indian or Alaska Native (non-Hispanic) | 0.4% (0.2-0.8) | 1.2% (0.0-6.2) | |
| | Another race / Unknown (non-Hispanic) | 0.2% (0.1-0.3) | 0.4% (0.0-1.4) | |
| | Two or more race groups selected (non-Hispanic) | 2.9% (2.3-3.5) | 1.3% (0.4-3.3) | |
| Education Level | Less than High School | 6.0% (5.2-6.9) | 4.8% (2.3-8.8) | 0.0344 |
| | High school graduate or GED | 15.9% (14.7-17.3) | 20.6% (14.4-27.9) | |
| | Some college or 2-year degree | 31.0% (29.4-32.7) | 38.1% (30.6-46.1) | |
| | 4-year college graduate or more | 47.0% (45.3-48.8) | 36.6% (29.1-44.5) | |
| Insurance Type | Medicare, Medicaid, Special Program | 30.9% (29.4-32.5) | 38.6% (31.3-46.3) | 0.0342 |
| | Commercial | 69.1% (67.5-70.6) | 61.4% (53.7-68.7) | |
| Income Level | < $35,000 | 20.2% (18.7-21.6) | 27.9% (20.3-36.5) | 0.0007 |
| | $35,000 - $75,000 | 31.6% (29.8-33.4) | 41.4% (32.5-50.7) | |
| | ≥ $75,000 | 48.3% (46.4-50.2) | 30.7% (22.6-39.8) | |

© 2025 Clennin M et al. *JAMA Network Open*.

| | | | | |
|---|---|---|---|---|
| **Marital Status** | Married / Living w/ Partner | 65.8% (64.1-67.5) | 46.5% (38.7-54.4) | <.0001 |
| | Previously Married | 13.8% (12.7-15.0) | 17.3% (11.9-23.8) | |
| | Never Married | 20.4% (18.9-21.8) | 36.2% (28.5-44.6) | |
| **Social Risk Factors** | | | | |
| Food Insecurity | | | | |
| | No Risk | 70.0% (68.3-71.6) | 56.8% (48.5-64.8) | 0.0006 |
| | Risk | 30.0% (28.4-31.7) | 43.2% (35.2-51.5) | |
| Housing Instability | | | | |
| | No Risk | 83.8% (82.4-85.0) | 74.0% (66.0-80.9) | 0.0020 |
| | Risk | 16.2% (15.0-17.6) | 26.0% (19.1-34.0) | |
| Financial Strain | | | | |
| | No Risk | 56.8% (55.1-58.6) | 37.4% (29.5-45.7) | <.0001 |
| | Risk | 43.2% (41.4-44.9) | 62.6% (54.3-70.5) | |
| Any Financial strain, Housing instability, and/or food insecurity [3] | | | | |
| | No Risk | 49.7% (47.9-51.5) | 40.7% (33.3-48.4) | 0.0211 |
| | Risk | 50.3% (48.5-52.1) | 59.3% (51.6-66.7) | |
| **Health Status** | | | | |
| Self-rated physical health | Excellent / Very Good | 48.3% (46.5-50.1) | 43.2% (35.7-50.9) | 0.1109 |
| | Good | 37.8% (36.0-39.5) | 37.2% (29.8-45.2) | |
| | Fair / Poor | 13.9% (12.7-15.2) | 19.6% (13.8-26.6) | |
| Self-rated mental health | Excellent / Very Good | 55.9% (54.1-57.7) | 48.3% (40.5-56.1) | 0.1232 |
| | Good | 29.6% (28.0-31.3) | 32.6% (25.3-40.6) | |
| | Fair / Poor | 14.5% (13.3-15.8) | 19.1% (13.2-26.3) | |
| **Charlson Comorbidity Index** | None, 0 | 60.8% (59.0-62.5) | 63.4% (55.7-70.6) | 0.8950 |
| | Mild, 1-2 | 28.5% (26.9-30.1) | 26.0% (19.7-33.2) | |
| | Moderate, 3-4 | 7.6% (6.7-8.5) | 7.8% (4.3-12.7) | |
| | Severe >=5 | 3.2% (2.7-3.7) | 2.8% (0.8-6.8) | |
| **Health Service Utilization** | | | | |
| | | | | |

© 2025 Clennin M et al. *JAMA Network Open*.

| | | | | |
|---|---|---|---|---|
| Emergency Department Encounter, Count *12 months prior to survey completion | None, 0 | 87.2% (85.9-88.3) | 81.8% (74.5-87.8) | 0.0098 |
| | 1 | 9.6% (8.5-10.7) | 12.6% (7.6-19.2) | |
| | 2-5 | 3.1% (2.6-3.7) | 4.2% (1.7-8.5) | |
| | 6 or more | 0.2% (0.1-0.3) | 1.4% (0.1-6.1) | |
| Inpatient Hospitalization Encounter, Count *12 months prior to survey completion | | | | |
| | None, 0 | 95.7% (94.9-96.4) | 93.5% (87.8-97.1) | 0.1184 |
| | 1 | 3.2% (2.6-3.8) | 5.8% (2.4-11.6) | |
| | 2-5 | 1.1% (0.7-1.5) | 0.7% (0.1-2.0) | |
| | 6 or more | 0.0% (0.0-0.1) | 0.0% (0.0-0.8) | |

%, precent; CI, confidence interval; SD, standard deviation

[1] Unweighted n = 9,785 patients; Survey respondents that completed the initial survey were eligible (n=10,226). NSNS data were merged with electronic health records (EHR) using a unique member ID (100% match). EHR data from January 2019 through July 2023 were extracted and included the following domains: acute care utilization, membership enrollment periods, health outcomes, and demographics. After exclusions, the final analytic study cohort included 9,785 adults with complete data. Exclusions ranged from 3.1% – 6.2% of the regional samples.

[2] Unweighted n = 489 patients; Exclusion criteria included: 1) those not enrolled in health plan at time of the first survey completion (allowing time for utilization to occur; 30-day lapse in coverage permitted) (n=338); and 2) those with incomplete data for social risk variables or covariates (n=151). Exclusions ranged from 3.1% – 6.2% of the regional samples.

[3] Any social risk defined as screening positive for one or more social risk factors including housing instability, food insecurity, and/or financial strain (Supplemental Table 1).

© 2025 Clennin M et al. *JAMA Network Open*.

**eTable 3**. Rate of Acute Care Utilization (ED Visits and Hospitalizations) by level of social risk exposure among weighted analytic cohort (supporting Figure 1).[2]

| Social Risk Factors | Social Risk Level | Hospitalizations Rate (95% CI) [1] | ED Visits Rate (95% CI) [1] |
|---|---|---|---|
| | | | |
| Any Financial Strain, Housing Instability, Food Insecurity | Social Risk | 40.6 (35.2 – 46.9) | 115.0 (104.3 – 126.8) |
| | None (ref) | 33.8 (29.2 – 39.1) | 86.9 (78.6 – 96.1) |
| | | | |
| Housing Instability | Social Risk | 47.2 (36.4 – 61.1) | 134.6 (115.4 – 157.0) |
| | None (ref) | 35.4 (31.7 – 39.4) | 94.5 (87.3 – 102.3) |
| | | | |
| Food Insecurity | Social Risk | 44.4 (36.6 – 53.9) | 124.6 (110.2 – 140.8) |
| | None (ref) | 34.2 (30.3 – 38.7) | 91.0 (83.7 – 99.0) |
| | | | |
| Financial Strain | Social Risk | 40.2 (34.2 – 47.1) | 118.5 (107.2 – 131.0) |
| | None (ref) | 35.0 (30.5 – 40.1) | 87.9 (80.0 – 96.7) |

[1] Rate per 1,000 person years
[2] Unweighted n = 9,785 adults

© 2025 Clennin M et al. *JAMA Network Open*.

**eTable 4**. Sensitivity Analysis: Adjusted hazard ratios (HR) of emergency department (ED) visits by social risk factors; with and without race and ethnicity included in final adjusted model.[1]

| Social Risk Factors | Social Risk Level | ED Visits | |
|---|---|---|---|
| | | **Adjusted Model** (presented in manuscript) | **Adjusted Model -** Race and Ethnicity Removed |
| | | **HR (95% CI)** | **HR (95% CI)** |
| Any Financial Strain, Housing Instability, Food Insecurity | Social Risk | **1.21 (1.03 – 1.41)** | **1.21 (1.04 – 1.41)** |
| | None | 1.00 (Ref) | 1.00 (Ref) |
| | | | |
| Financial Strain | Social Risk | **1.20 (1.03 – 1.39)** | **1.20 (1.03 – 1.40)** |
| | None | 1.00 (Ref) | 1.00 (Ref) |
| | | | |
| Housing Instability | Social Risk | **1.31 (1.09 – 1.57)** | **1.30 (1.09 – 1.56)** |
| | None | 1.00 (Ref) | 1.00 (Ref) |
| | | | |
| Food Insecurity | Social Risk | **1.20 (1.02 – 1.40)** | **1.20 (1.03 – 1.40)** |
| | None | 1.00 (Ref) | 1.00 (Ref) |
| | | | |

[1] Model adjusted for individual-level sociodemographic factors (age group, sex, race and ethnicity, education, marital status) and health status factors (self-rated physical health, comorbidities). Covariates excluded from final analytic model after collinearity testing include insurance type, income level, employment status, self-reported mental health status.

CI, confidence intervals; HR, hazard ratios; ED emergency department

© 2025 Clennin M et al. *JAMA Network Open*.

**eTable 5**. Sensitivity Analysis: Adjusted hazard ratios (HR) of hospitalizations by social risk factors; with and without race and ethnicity included in final adjusted model.[1]

| Social Risk Factors | Social Risk Level | Hospitalizations | |
|---|---|---|---|
| | | **Adjusted** (presented in manuscript) | **Adjusted Model -** Race and Ethnicity Removed |
| | | **HR (95% CI)** | |
| Any Financial Strain, Housing Instability, Food Insecurity | Social Risk | 1.05 (0.84 – 1.32) | 1.02 (0.82 – 1.28) |
| | None (ref) | 1.00 (Ref) | 1.00 (Ref) |
| | | | |
| Financial Strain | Social Risk | 1.01 (0.81 – 1.26) | 0.99 (0.79 – 1.23) |
| | None (ref) | 1.00 (Ref) | 1.00 (Ref) |
| | | | |
| Housing Instability | Social Risk | 1.28 (0.94 – 1.74) | 1.24 (0.91 – 1.68) |
| | None (ref) | 1.00 (Ref) | 1.00 (Ref) |
| | | 1.00 (Ref) | 1.00 (Ref) |
| Food Insecurity | Social Risk | 1.05 (0.81 – 1.35) | 1.02 (0.79 – 1.30) |
| | None (ref) | 1.00 (Ref) | 1.00 (Ref) |
| | | | |

[1] Model adjusted for individual-level sociodemographic factors (age group, sex, race and ethnicity, education, marital status) and health status factors (self-rated physical health, comorbidities). Covariates excluded from final analytic model after collinearity testing include insurance type, income level, employment status, self-reported mental health status.

CI, confidence intervals; HR, hazard ratios;

© 2025 Clennin M et al. *JAMA Network Open*.

**eFigure 1**. CONSRT Diagram, unweighted cohort[1]

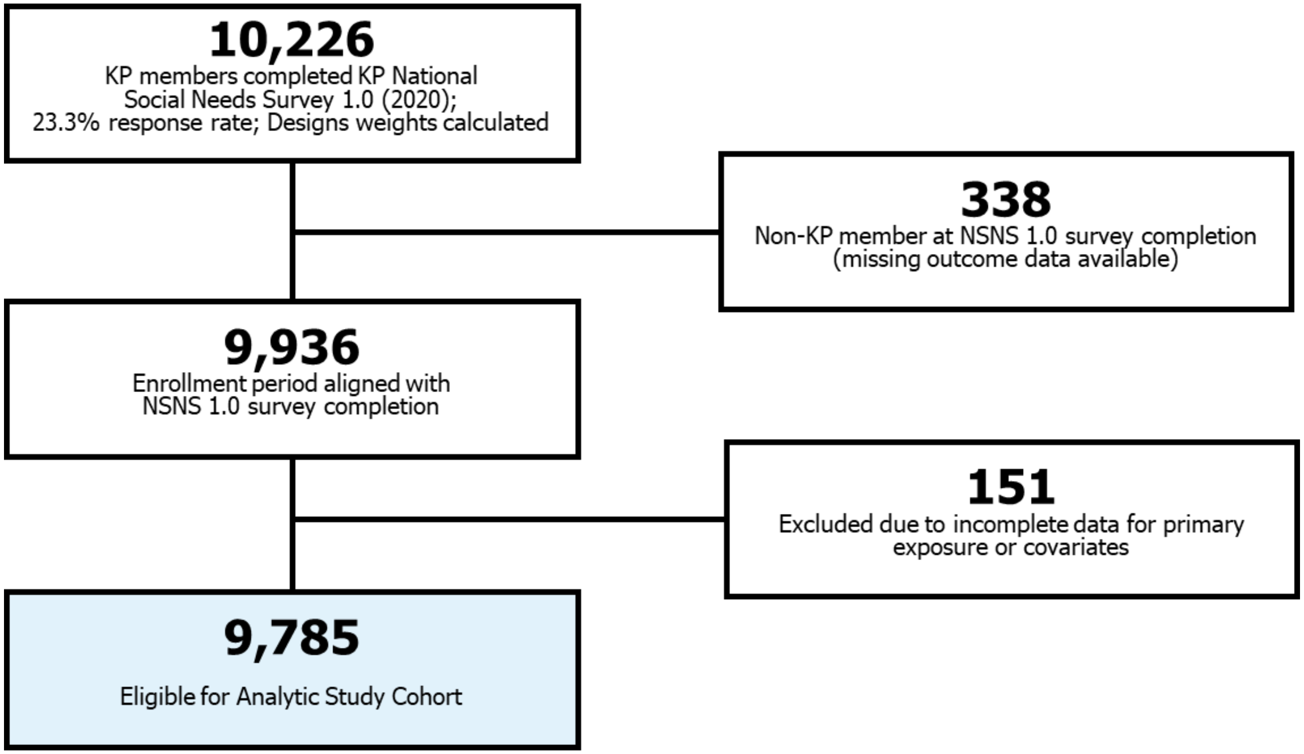


Notes.

[1] 1.0 survey wave administered between January-September 2020, 9,785 respondents met inclusion criteria and were included in the analytic study cohort (unweighted sample). Approximately two years later, 2.0 survey wave was administered between June 2022-February 2023. Of the 9,785 survey respondent meeting eligibility criteria, 6,003 (61.3%) respondents completed the 2.0 survey (unweighted sample).

© 2025 Clennin M et al. *JAMA Network Open*.
