# Supplementary material for: Social Risk and Acute Health Care Utilization Among Insured Adults
Source: JAMA Netw Open. 2025 Apr 1;8(4):e254253. doi: 10.1001/jamanetworkopen.2025.4253 (PMC11962667; doi:10.1001/jamanetworkopen.2025.4253)
Supplement: Supplement 2. — Data Sharing Statement [file jamanetwopen-e254253-s002.pdf]

Data Sharing Statement

Clennin. Social Risk and Acute Health Care Utilization Among Insured Adults. *JAMA Netw Open*. Published April 01, 2025. doi:10.1001/jamanetworkopen.2025.4253

Data
Data available: No

Additional Information
Explanation for why data not available: Deidentified survey response data are available upon reasonable request. Clinical data are not publicly available. Please contact the corresponding author for further information.
